# Supplementary material for: Genome wide analysis revealed conserved domains involved in the effector discrimination of bacterial type VI secretion system
Source: Commun Biol. 2023 Nov 24;6:1195. doi: 10.1038/s42003-023-05580-w (PMC10673891; doi:10.1038/s42003-023-05580-w)
Supplement: Supplementary file 2 — Supplementary Information [file 42003_2023_5580_MOESM2_ESM.pdf]

Genome wide analysis revealed conserved domains involved in the effector discrimination of bacterial type VI secretion system

Caihong Wang,[1,†] Mingxing Chen,[2,†] Yuhan Shao[1], Mengyuan Jiang,[1] Quanjie Li,[3] Lihong Chen,[2] Yun Wu,[1] Shan Cen,[3,4] Nicholas R. Waterfield,[5] Jian Yang,[2,*] and Guowei Yang[1,]**

[1] Beijing Institute of Tropical Medicine, Beijing Friendship Hospital, Capital Medical University, Beijing 100050, China

[2] NHC Key Laboratory of Systems Biology of Pathogens, National Institute of Pathogen Biology, Chinese Academy of Medical Sciences & Peking Union Medical College, Beijing 102629, China

[3] Institute of Medicinal Biotechnology, Chinese Academy of Medical Sciences & Peking Union Medical College, Beijing 100050, China

[4] CAMS Key Laboratory of Antiviral Drug Research, Peking Union Medical College, Chinese Academy of Medical Sciences, Beijing 100730, China

[5] Warwick Medical School, Warwick University, Coventry CV4 7AL, UK

[†] These authors contributed equally

*Corresponding author. Tel: +86 10 67875146; E-mail: yangj@ipbcams.ac.cn

** Corresponding author. Tel: +86 10 63139030; E-mail: yangguowei@hotmail.com

Supplementary Figure 1

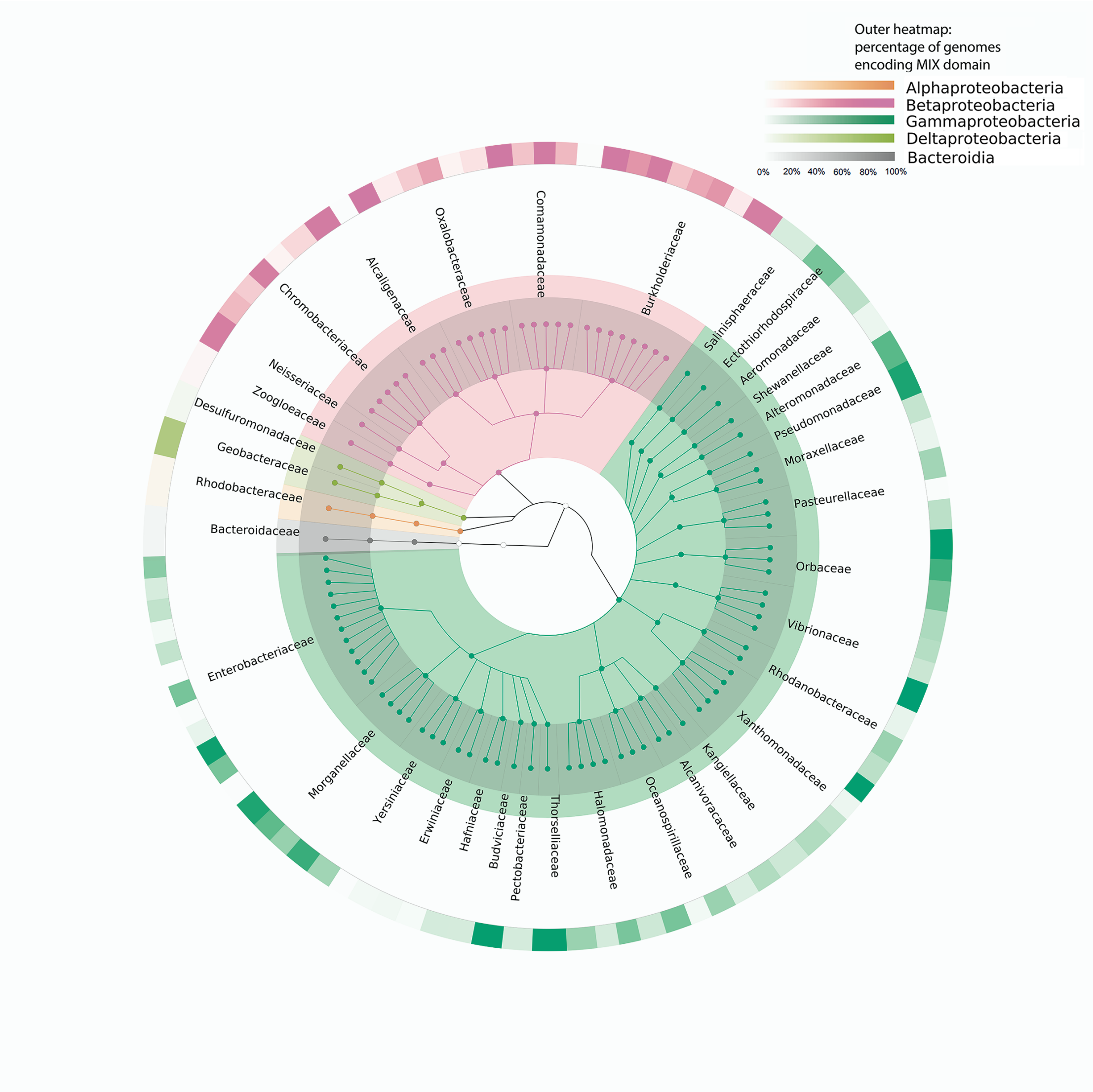

Supplementary Fig. 1: The taxonomic distribution of 7,208 MIX domains encoded within the *vgrG* loci.

Only taxa with genomes encoding MIX-encoding *vgrG* loci are shown for brevity. Genomes without known assigned genus are excluded. The circles represent phylum, class, order, family and genus from inner to outer, and are color-coded by phylum/class (key). The family names are given outside the taxonomic tree. The outer heatmap represents the percentage of genomes encoding the MIX domain for each genus (key).

Supplementary Figure 2

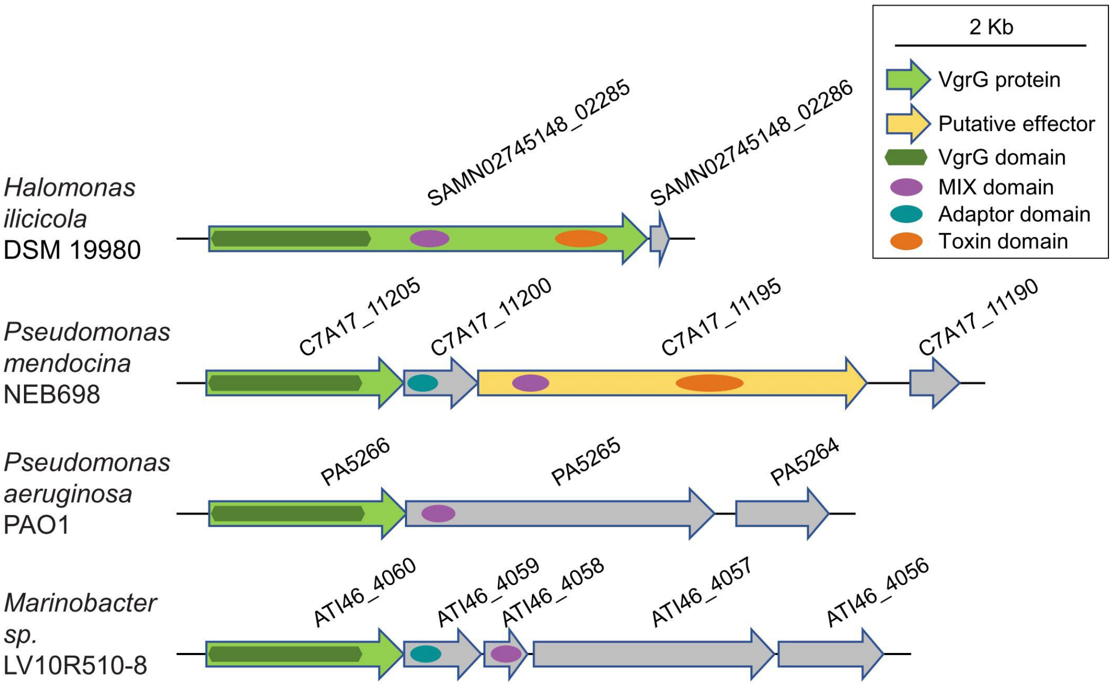

Supplementary Fig. 2: Representative loci of the MIX domain.

For brevity, only the genomic region downstream of the *vgrG* gene was shown for each locus. The locations of VgrG domain, MIX domain, adaptor and toxin domains were highlighted (key). Gene ID was indicated above each gene arrow.

Supplementary Figure 3

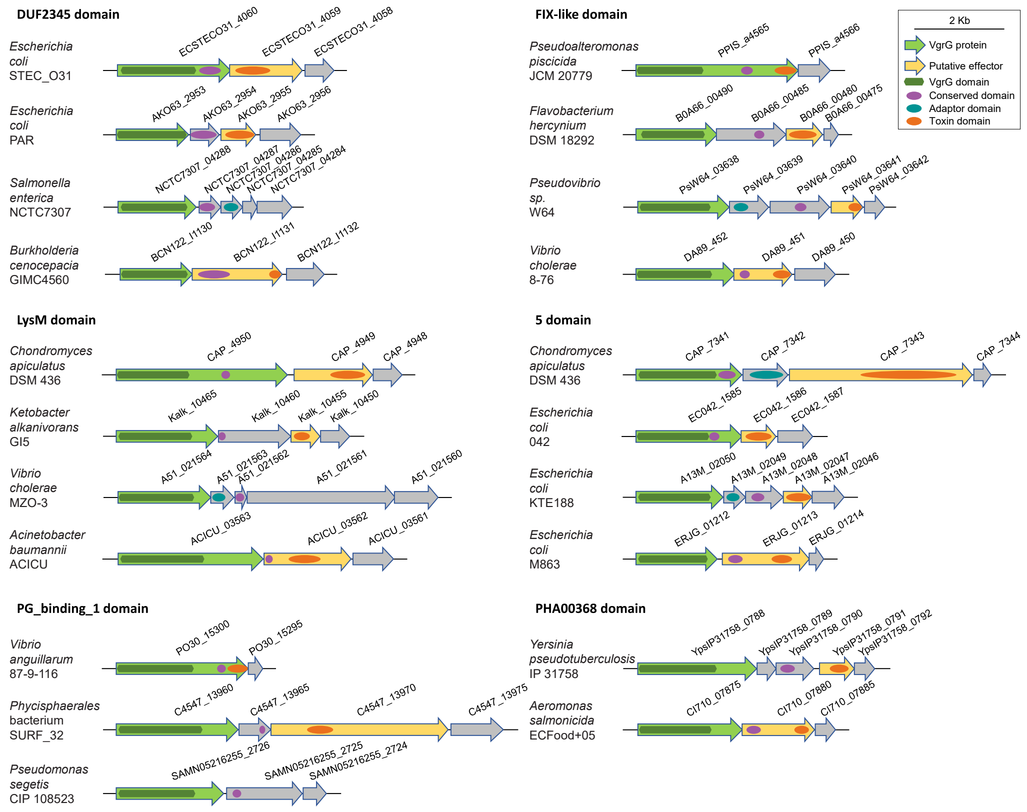

Supplementary Fig. 3: Representative genetic organization of the six conserved domains with multiple encoding forms.

For brevity, only the genomic region downstream of the *vgrG* gene was shown for each locus. The locations of VgrG domain, MIX domain, adaptor and toxin domains were highlighted (key). Gene ID was indicated above each gene arrow.

Supplementary Figure 4

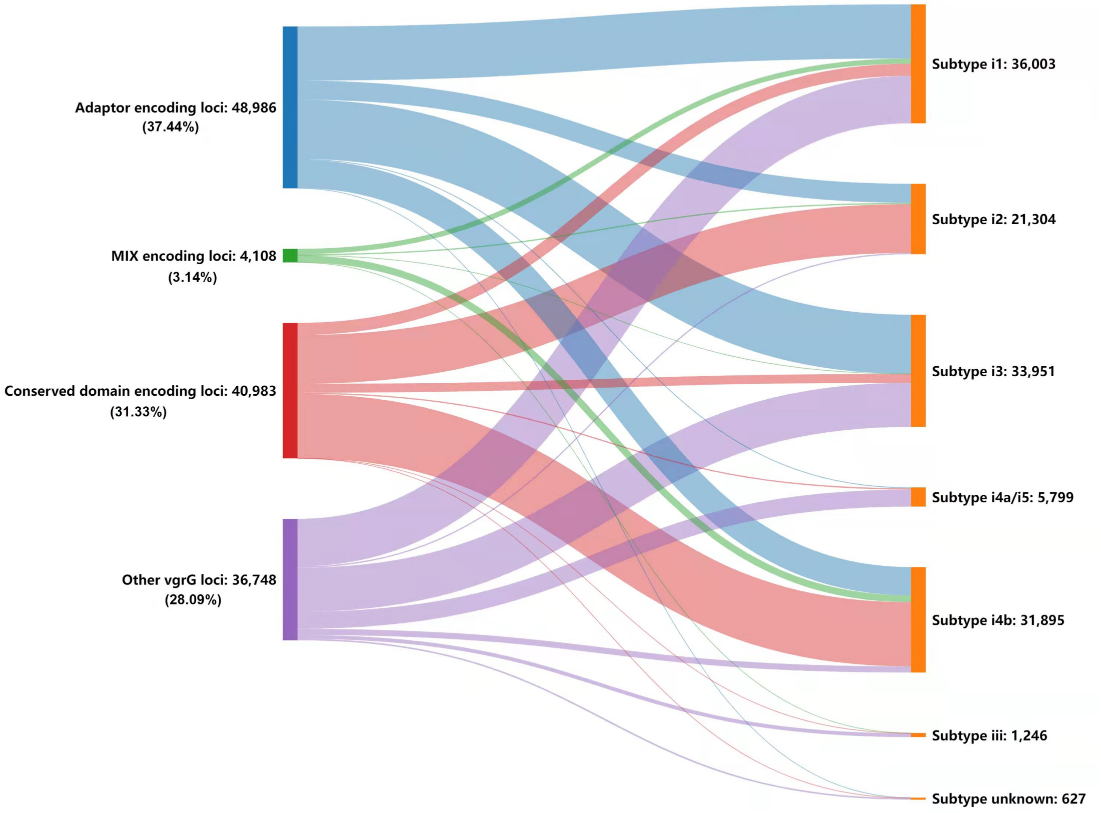

Supplementary Fig. 4: A Sankey diagram showing the relationship between the adaptor/MIX/conserved domains encoding *vgrG* loci (left) and the known subtypes of T6SS (right).

Only the three reported adaptor domains (i.e. DcrB, DUF4123 and DUF2169), the MIX domain, and the six conserved domains identified in current study among all of the 130,825 loci analyzed. The *vgrG* loci calculation was done progressively in the order of adaptors, MIX domain, and conserved domains.

Supplementary Figure 5

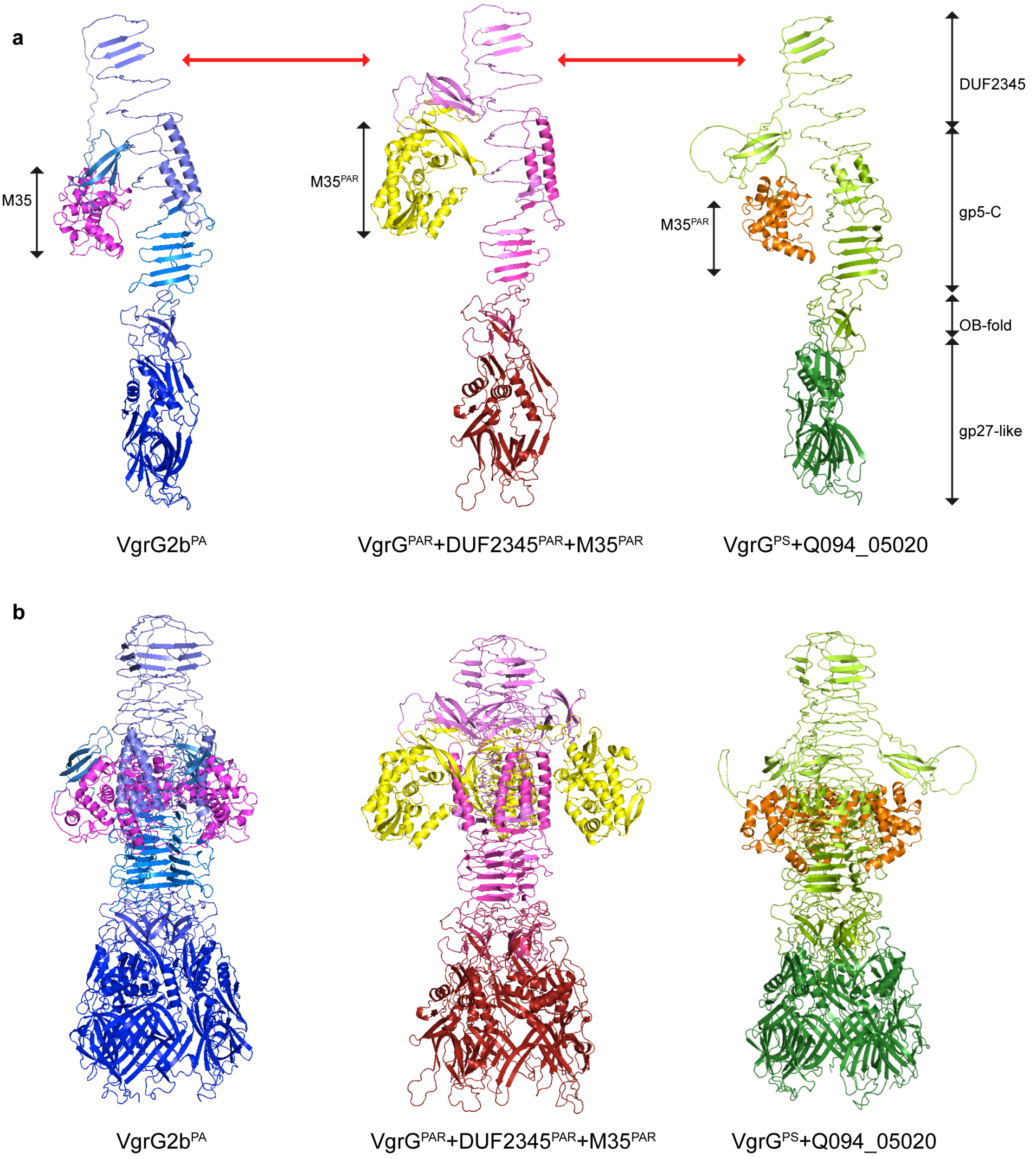

Supplementary Fig. 5: Models of the VgrG proteins

a Comparison of structure models of VgrG2b^{PA} in *P. aeruginosa* PAO1 (left), the VgrG^{PAR}-DUF2345^{PAR}-M35^{PAR} complex in *E. coli* ^{PAR} (middle) and the VgrG^{PS}-Q094_05019^{PS} complex in *P. aeruginosa* PS42 (right). Red arrow: DUF2345 forms three-stranded β-prism to connect gp5-C like and M35 domain. **b** Structure models of the full VgrG2b^{PA} trimer (left), the VgrG^{PAR}-DUF2345^{PAR}-M35^{PAR} complex (middle) and the VgrG^{PS}-Q094_05019^{PS} complex (right).

Supplementary Figure 6

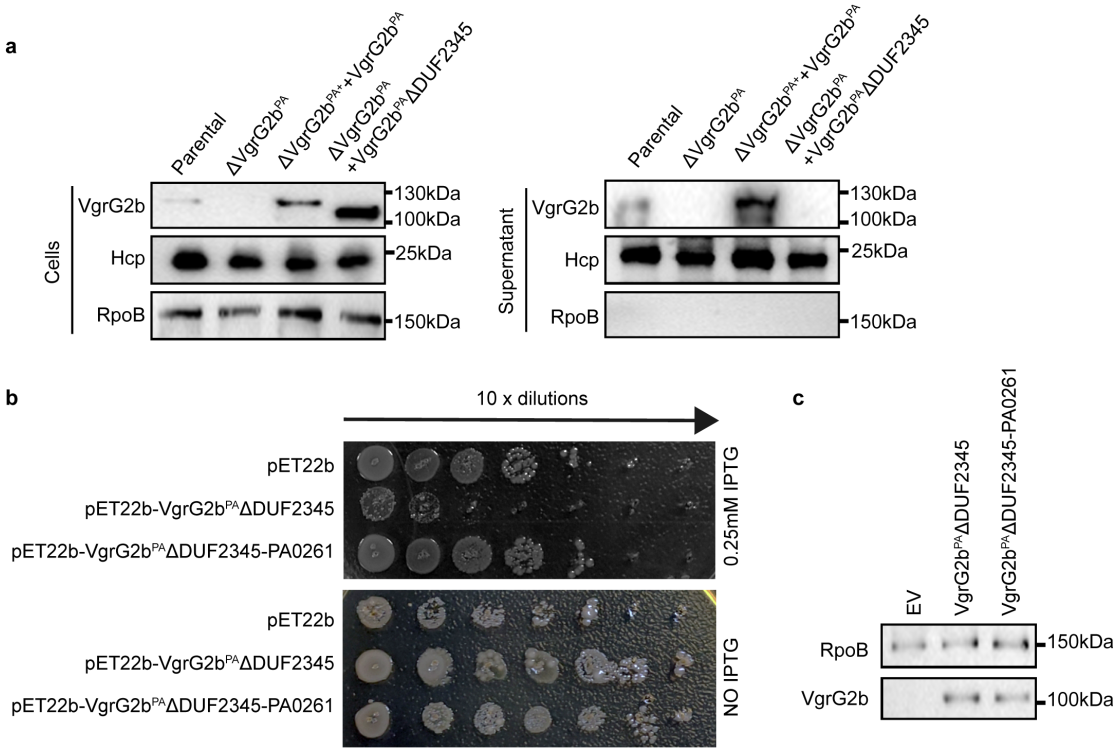

Supplementary Fig. 6: DUF2345 domain assist the delivery of T6SS effector.
a Immunoblots demonstrating the secretion of VgrG2b^{PA} by the H2-T6SS. Anti-RpoB is lysis control. **b** Survival of *E. coli* expressing the truncated mutant VgrG2b^{PA}ΔDUF2345 in pET22b. Ten-fold serial dilutions of cultures were spotted on LB agar containing the stated concentrations of IPTG and grown for 24h. The image is representative of three independent experiments. **c** Immunoblots demonstrating the expression of truncated mutant VgrG2b^{PA}ΔDUF2345 in *E. coli*. Anti-RpoB is lysis control.

Supplementary Figure 7

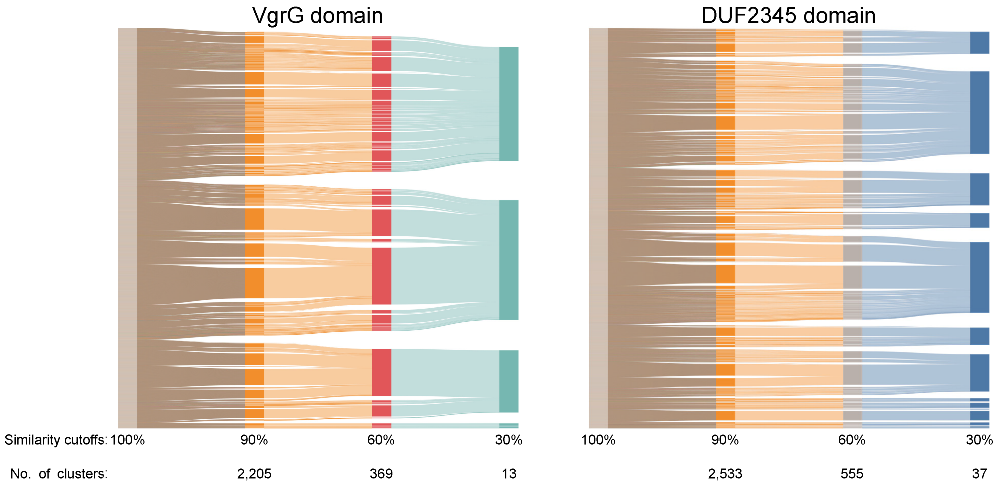

Supplementary Fig. 7: Iterative clustering of VgrG and DUF2345 domain sequences of *vgrG* loci.

The VgrG domains were clustered into 2,205, 369 and 13 clusters at the similarity cutoffs of 90%, 60% and 30%, respectively (left). The DUF2345 domains were clustered into 2,533, 555 and 37 clusters at the similarity cutoffs of 90%, 60% and 30%, respectively (right). The protein sequence clustering procedures were conducted by CD-HIT.

Supplementary Figure 8

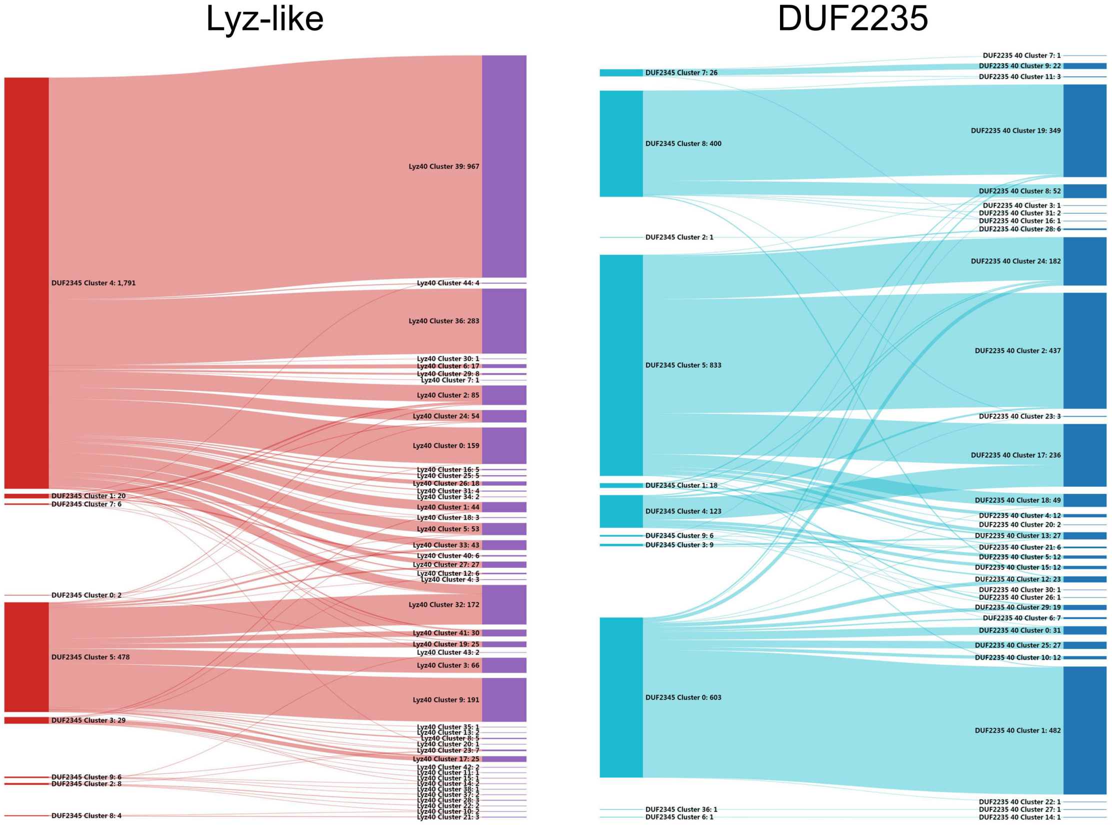

Supplementary Fig. 8: The relationship between DUF2345 clusters and downstream toxin clusters.

A total of 2,344 and 2,021 loci were included for the clustering of Lyz-like (left) and DUF2235 (right) domains. For each panel, the DUF2345 clusters were shown on the left and the toxin clusters were shown on the right. The protein sequence clustering was conducted by CD-HIT. The number of sequences in each cluster is given after the cluster name.

Supplementary Figure 9

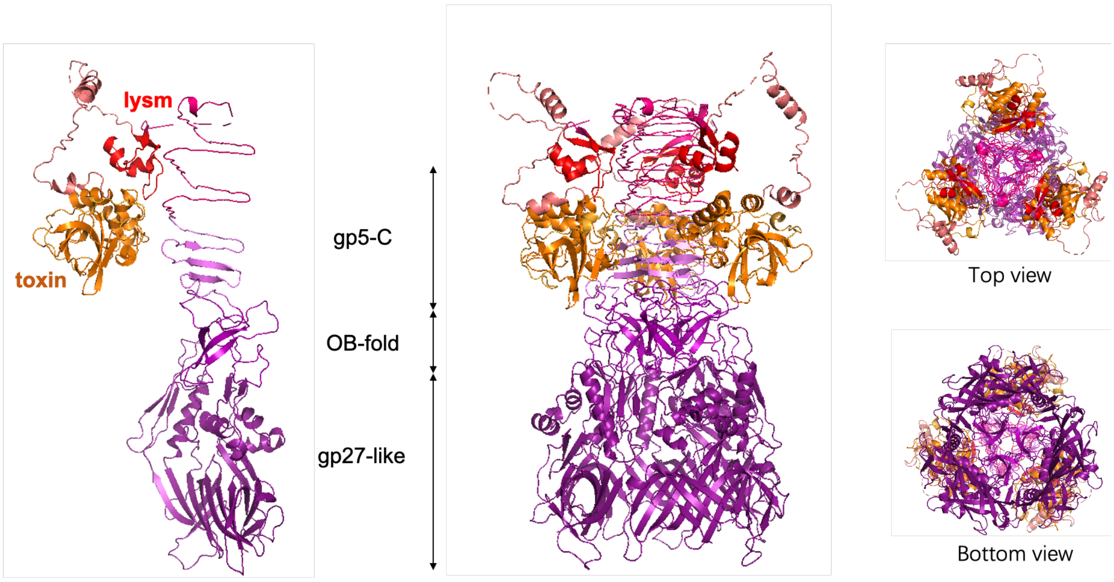

Supplementary Fig. 9: Model of the complex of BRPE_05230 and BRPE_05220 proteins in *Burkholderia* sp. RPE67.

Structure model of the $VgrG^{RPE}$-$LysM^{RPE}$-$NLPC\_P60^{RPE}$ complex monomer (left) and the trimer complex (right).

Supplementary Figure 10

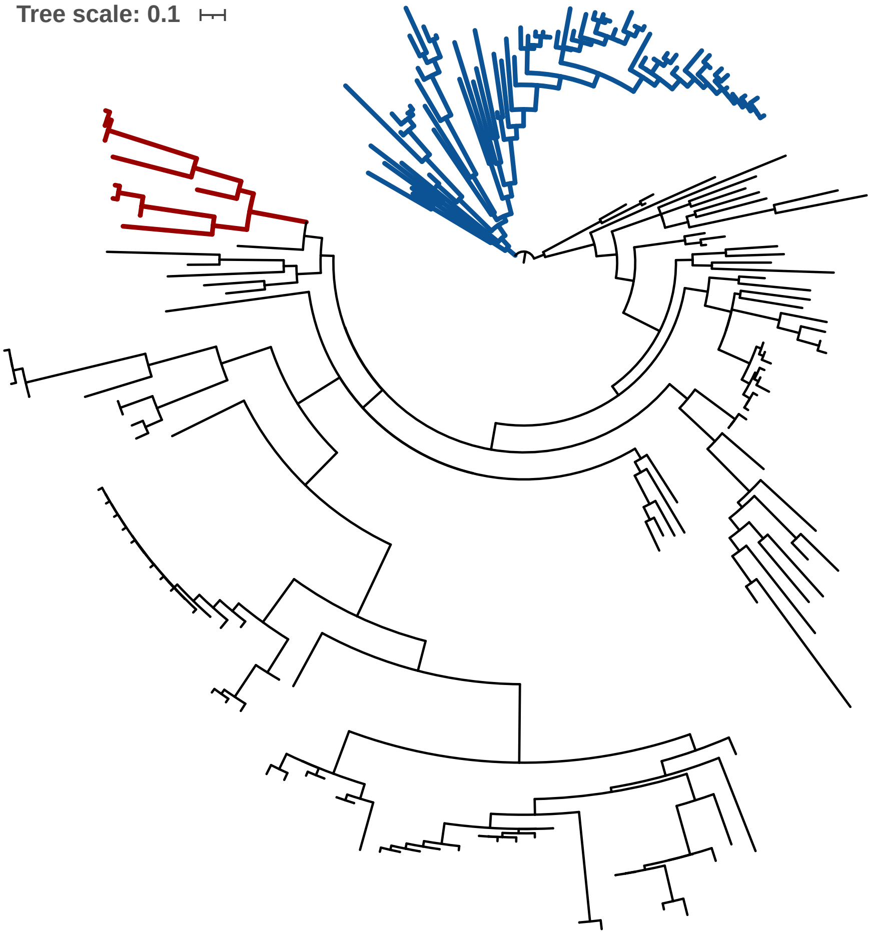

Supplementary Fig. 10: Phylogenetic tree of LysM domain.

A maximum-likelihood tree of the 2,415 VgrG-downstream, 737 eCIS-related (available from dbeCIS) and 19 phage-encoded LysMs based on the sequences of their LysM domain (CDD accession: cl21525). Identical sequences within each group were removed to retain only one representative for brevity. The tree was constructed by FastTree under Whelan Goldman (WAG) models with gamma optimization. The eCIS-related and phage-encoded LysMs are highlighted by blue and red branches, respectively. The tree scale represents substitutions per site.

Supplementary Figure 11

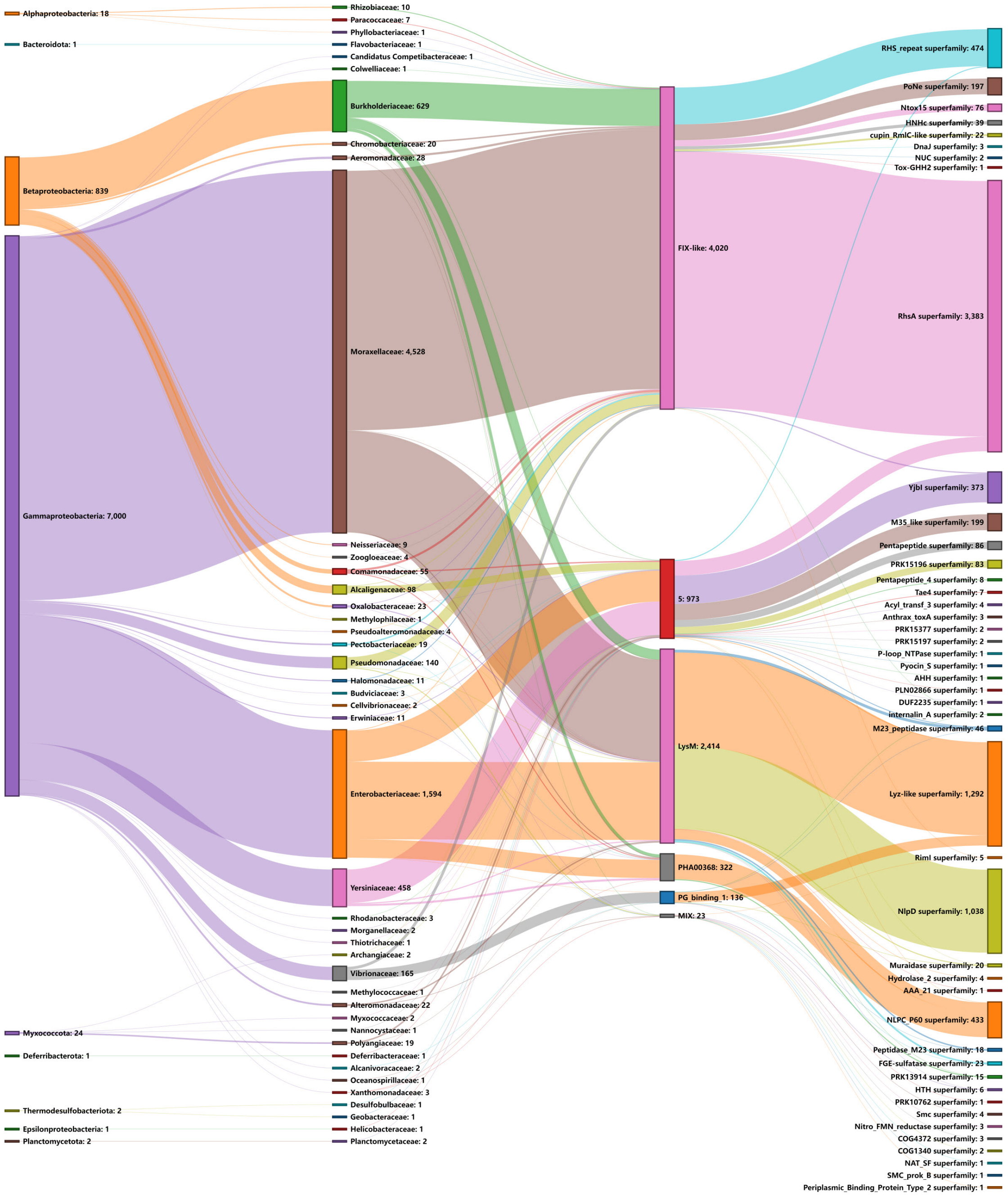

Supplementary Fig. 11: Sankey diagram showing the relationship between bacterial phylum/class, family, the VgrG-related conserved domains and the downstream toxin domain families (from left to right).

For brevity DUF2345 was excluded as it was already analyzed in Figure 4. Loci from genomes without necessary taxa information were excluded. The number of sequences involved in each node is given after the node name.

Supplementary Table 1. MIX and six conserved domain/proteins encoded within *vgrG* loci

**Conserved domain (accession)**	**Adjacent to known toxin gene/domains**			**Not adjacent to known toxin gene/domains**	**Total**
	Encoded at the C-terminus of *vgrG*	Encoded as a stand-alone gene	Encoded at the N-terminus of toxin gene		
MIX (cl41762)		17	10	7,181	7,208
DUF2345 (cl01733)	17,916	89		34,272	52,277
FIX-like (cl41761)	5	60	3,963	1,330	5,358
LysM (cl21525)	2	67	2,346	2,213	4,628
5 (cl33691)	976	5	1	866	1,848
PG_binding_1 (cl38043)	134	5	4	533	676
PHA00368 (cl30808)		6	316	39	361

*** Data based on the 130,825 *vgrG* loci analyzed in this work**

Supplementary Table 2. Bacterial strains and plasmids used in this study.

Bacterial strains and plasmids	SOURCE	IDENTIFIER
Strains		
*Escherichia coli Trans*5α	TransGen Biotech	CD201
BL21(DE3) pLysS	TransGen Biotech	CD701
Escherichia coli S17	Lab stock	N/A
PAO1	Lab stock	N/A
PAO1ΔPA0262	This paper	N/A
PAO1ΔPA0262ΔPA0261	This paper	N/A
PAO1ΔPA0262+PA0262	This paper	N/A
PAO1ΔPA0262+ PA0262ΔDUF2345	This paper	N/A
plasmid		
pET22 b(+)	Lab stock	N/A
pET22b-PA0262	This paper	N/A
pET22b-PA0261-PA0262	This paper	N/A
pET22b-PA0262ΔM35	This paper	N/A
pET22b-AKO63_2955	This paper	N/A
pET22b-AKO63_2955-2956	This paper	N/A
pET22b-AKO63_2954	This paper	N/A
pME6032	Lab stock	N/A
pME6032-PA0262	This paper	N/A
pME6032-PA0262ΔDUF2345	This paper	N/A
pETduet	Lab stock	N/A
RSFduet	Lab stock	N/A
pETduet-Myc-VgrG	This paper	N/A
RSFduet-Flag-DUF	This paper	N/A
RSFduet-M35-Stag	This paper	N/A
RSFduet-Flag-DUF-M35-Stag	This paper	N/A

RSFduet-Flag-M35	This paper	N/A
RSFduet-DUF-Stag	This paper	N/A
RSFduet-Flag-M35-DUF-Stag	This paper	N/A
pETduet-VgrG^{G15}-Myc	This paper	N/A
pETduet-LysM^{G15}-strep	This paper	N/A
pETduet-VgrG^{G15}-LysM^{G15}	This paper	N/A
RSFduet-Kalk_10455^{G15}-flag	This paper	N/A
RSFduet-Kalk_10450^{G15}-Stag	This paper	N/A
RSFduet-Kalk_10455^{G15}-Kalk_10450^{G15}	This paper	N/A
pETduet-VgrG^{RPE}-Myc	This paper	N/A
RSFduet-BRPE_05220^{RPE}-flag	This paper	N/A
RSFduet-BRPE_05220^{RPE}ΔLysM-flag	This paper	N/A
RSFduet-BRPE_05230-Stag	This paper	N/A
RSFduet-BRPE_05220^{RPE}ΔLysM-BRPE_05230	This paper	N/A

Supplementary Table 3. Software and algorithms used in this study.

Software and Algorithms	SOURCE	IDENTIFIER
SecReT6	(Li et al, 2015)[1]	http://db-mml.sjtu.edu.cn/SecReT6/
CDD	(Lin et al, 2020)[2]	https://www.ncbi.nlm.nih.gov/cdd
dbeCIS	(Chen et al, 2019)[3]	http://www.mgc.ac.cn/dbeCIS/
Clustal-Omega	(Sievers et al, 2011)[4]	https://www.ebi.ac.uk/Tools/msa/clustalo/
FastTree v2.1	(Price et al, 2010)[5]	http://meta.microbesonline.org/fasttree/
iTOL	(Letunic & Bork, 2021)[6]	https://itol.embl.de/
CD-HIT v4.6.5	(Li & Godzik, 2006)[7]	http://weizhong-lab.ucsd.edu/cd-hit/
SankeyMATIC	-	https://sankeymatic.com/
VFDB	(Liu et al, 2022)[8]	http://www.mgc.ac.cn/VFs
HMMER3 v3.1b2	(Mistry et al, 2013)[9]	http://hmmer.org/
dbVgrG	This paper	http://www.mgc.ac.cn/dbVgrG/

Supplementary Table 4. Primers used in this study.

Primer[a]	Sequence (5'-3')[b,c]
pETduet-Myc-VgrG-F1	TCGAGCTCGGCGCGCCTGCAGGTCGACGAGCAGAAACTCATCTCTGAAGAGGATCTG
pETduet-Myc-VgrG-F	TCTGAAGAGGATCTGggaggtggaggatccATGCAGGACGGCGCATTCAG
pETduet-Myc-VgrG-R	CTGTTCGACTTAAGCATTATGCGGCCGCCTACGCATCAGTGGACAGCGA
RSFduet-Flag-DUF-F1	TAATAAGGAGATATACCATGGGCGATTACAAGGATGACGACGATAAG
RSFduet-Flag-DUF-F	ATGACGACGATAAGggaggtggaggatccTTGCAGCAGAGTGTAAAAG
RSFduet-Flag-DUF-R	GTTCGACTTAAGCATTATGCGGCCGCTTACTCATGCACGCCTCCCAAT
RSFduet-M35-Stag-F	TATAAGAAGGAGATATACATATGAGTAATGATAGAGGTAGTTCA
RSFduet-M35-Stag-R	CAGCGGTTTC TTTACCAGACTCGAGATCTATTTCAAAATA ACGTTCA
RSFduet-Flag-M35-F1	TAATAAGGAGATATACCATGGGCGATTACAAGGATGACGACGATAAG
RSFduet-Flag-M35-F	ATGACGACGATAAGggaggtggaggatccAGTAATGATAGAGGTAGTTCA
RSFduet-Flag-M35-R	GTTCGACTTA AGCATTATGCGGCCGCTTAATCTATTTCAAAATAACGTTCA
RSFduet-DUF-Stag-F	TATAAGAAGGAGATATACATATGTTGCAGCAGAGTGTAAAAG
RSFduet-DUF-Stag-R	CAGCGGTTTCTTTACCAGACTCGAGCTCATGCACGCCTCCCAAT
pET22b-AKO63_2955-F	GAATTAATTCGGATCCGAATTCATGAGTAATGATAGAGGTAG
pET22b-AKO63_2955-R	TGGTGCTCGAGTGCGGCCGCAAGCTTTTAATCTATTTCAAAATAACGT
pET22b-AKO63_2955-2956-ov-F	CGTTATTTTGAAATAGATTAATTATTGATCTCCATTTGCTGATC
pET22b-AKO63_2955-2956-ov-R	GATCAGCAAATGGAGATCAATAATTAATCTATTTCAAAATAACG
pET22b-AKO63_2955-2956 -R	TGGTGCTCGAGTGCGGCCGCAAGCTTTCATTGCATCGACGAATACAGG
pET22b-AKO63_2954-F	GAATTAATTCGGATCCGAATTCTTGCAGCAGAGTGTAAAAGATCT
pET22b-AKO63_2954-R	TGGTGCTCGA GTGCGGCCGC AAGCTTTTAC TCATGCACGC CTCCCAATA
pME6032-PA0262-F1	ACAATTTCACACAGGAAACAGAATTCATGGATTACAAGGATGACGACGATAAG
pME6032-PA0262-F	GATTACAAGGATGACGACGATAAGATGCGTCAAAGGGACCTGAAATTC
pME6032-PA0262-R	ACTGATCCGCTAGTCCGAGGCCTCGAGTCAGTATCCCGTTGGGAAGTTTTTC
PA0262ΔDUF-lin-F	GTGCGCGACCTCGGCCACCTGGAAGCCGAGCTGCCGC
PA0262ΔDUF-lin-R	GCGGCAGCTCGGCTTCCAGGTGGCCGAGGTCGCGCAC
pET22b-PA0262-F	GAATTAATTCGGATCCGAATTCATGCGTCAAAGGGACCTGAAATTC
pET22b-PA0262-R	TGGTGCTCGAGTGCGGCCGCAAGCTTTCAGTATCCCGTTGGGAAGTTTTTC

pET22b-PA0261-PA0262-F	GAATTAATTCGGATCCGAATTCATGCGTCAAAGGGACCTGAAATTC
pET22b-PA0261-PA0262-R	TGGTGCTCGAGTGCGGCCGCAAGCTTTCAT GGCTTCTCTC CTTGC
pET22b-PA0262ΔM35-F	GAATTAATTCGGATCCGAATTCATGCGTCAAAGGGACCTGAAATTC
pET22b-PA0262ΔM35-R	TGGTGCTCGAGTGCGGCCGCAAGCTTTTTCGTGTGTTTCATATCGACCTT G
PK18-PA0262-up-F	ATTCGAGCTCGGTACCCGGGGATCCTCACCGAGGACTCGGTCGGCAA
PK18-PA0262-up-R	GAGCAAGGTC TTGCATTTCATTGGTCAGTATTGACGCATGGATCGTTCCT TGTTCCT
PK18-PA0262-down-F	AGGAACAAGGAACGATCCATGCGTCAATACTGACCAATGAAATGCAAGACCTTGCTC
PK18-PA0262-down-R	CTTGCATGCCTGCAGGTCGACTCTAGAAGGTGATGGACTGGGCAGAGACG AT
NLPC_P60-F	ACCATCATCACCACAGCCAGGATCCGTCGGTTTGGGATCGGACATG
NLPC_P60-R	TTAAGCATTATGCGGCCGCAAGCTT

a F1 primers were used to add the tags into the genes by overlap extension PCR.

b Restriction sites displayed with underline.

c Linker sequences displayed in lowercase.

Reference:

1 Li, J. *et al.* SecReT6: a web-based resource for type VI secretion systems found in bacteria. *Environ Microbiol* **17**, 2196-2202, doi:10.1111/1462-2920.12794 (2015).

2 Lin, Y. *et al.* Genomic and transcriptomic alterations associated with drug vulnerabilities and prognosis in adenocarcinoma at the gastroesophageal junction. *Nat Commun* **11**, 6091, doi:10.1038/s41467-020-19949-6 (2020).

3 Chen, L. *et al.* Genome-wide Identification and Characterization of a Superfamily of Bacterial Extracellular Contractile Injection Systems. *Cell Rep* **29**, 511-521 e512, doi:10.1016/j.celrep.2019.08.096 (2019).

4 Sievers, F. *et al.* Fast, scalable generation of high-quality protein multiple sequence alignments using Clustal Omega. *Mol Syst Biol* **7**, 539, doi:10.1038/msb.2011.75 (2011).

5 Price, M. N., Dehal, P. S. & Arkin, A. P. FastTree 2--approximately maximum-likelihood trees for large alignments. *PLoS One* **5**, e9490, doi:10.1371/journal.pone.0009490 (2010).

6 Letunic, I. & Bork, P. Interactive Tree Of Life (iTOL) v5: an online tool for phylogenetic tree display and annotation. *Nucleic Acids Res* **49**, W293-W296, doi:10.1093/nar/gkab301 (2021).

7 Li, W. & Godzik, A. Cd-hit: a fast program for clustering and comparing large sets of protein or nucleotide sequences. *Bioinformatics* **22**, 1658-1659, doi:10.1093/bioinformatics/btl158 (2006).

8 Liu, B., Zheng, D., Zhou, S., Chen, L. & Yang, J. VFDB 2022: a general classification scheme for bacterial virulence factors. *Nucleic Acids Res* **50**, D912-

D917, doi:10.1093/nar/gkab1107 (2022).

9 Mistry, J., Finn, R. D., Eddy, S. R., Bateman, A. & Punta, M. Challenges in homology search: HMMER3 and convergent evolution of coiled-coil regions. *Nucleic Acids Res* **41**, e121, doi:10.1093/nar/gkt263 (2013).
