# Supplementary material for: Genome wide analysis revealed conserved domains involved in the effector discrimination of bacterial type VI secretion system
Source: Commun Biol. 2023 Nov 24;6:1195. doi: 10.1038/s42003-023-05580-w (PMC10673891; doi:10.1038/s42003-023-05580-w)
Supplement: Supplementary file 3 — Description of Additional Supplementary Files [file 42003_2023_5580_MOESM3_ESM.docx]

Description of Additional Supplementary Files

**File name:** Supplementary Data 1

**Description:** The source data behind the graphs in the paper

**File name:** Supplementary Data 2

**Description:** Dataset of bacterial genomes
